# Ecto-Nucleoside Triphosphate Diphosphohydrolase 2 Modulates Local ATP-Induced Calcium Signaling in Human HaCaT Keratinocytes

**DOI:** 10.1371/journal.pone.0057666

**Published:** 2013-03-11

**Authors:** Chia-Lin Ho, Chih-Yung Yang, Wen-Jie Lin, Chi-Hung Lin

**Affiliations:** 1 Institute of Microbiology and Immunology, National Yang-Ming University, Taipei, Taiwan; 2 Department of Education and Research, Taipei City Hospital, Taipei, Taiwan; University of Birmingham, United Kingdom

## Abstract

Keratinocytes are the major building blocks of the human epidermis. In many physiological and pathophysiological conditions, keratinocytes release adenosine triphosphate (ATP) as an autocrine/paracrine mediator that regulates cell proliferation, differentiation, and migration. ATP receptors have been identified in various epidermal cell types; therefore, extracellular ATP homeostasis likely determines its long-term, trophic effects on skin health. We investigated the possibility that human keratinocytes express surface-located enzymes that modulate ATP concentration, as well as the corresponding receptor activation, in the pericellular microenvironment. We observed that the human keratinocyte cell line HaCaT released ATP and hydrolyzed extracellular ATP. Interestingly, ATP hydrolysis resulted in adenosine diphosphate (ADP) accumulation in the extracellular space. Pharmacological inhibition by ARL 67156 or gene silencing of the endogenous ecto-nucleoside triphosphate diphosphohydrolase (NTPDase) isoform 2 resulted in a 25% reduction in both ATP hydrolysis and ADP formation. Using intracellular calcium as a reporter, we found that although NTPDase2 hydrolyzed ATP and generated sustainable ADP levels, only ATP contributed to increased intracellular calcium via P2Y2 receptor activation. Furthermore, knocking down NTPDase2 potentiated the nanomolar ATP-induced intracellular calcium increase, suggesting that NTPDase2 globally attenuates nucleotide concentration in the pericellular microenvironment as well as locally shields receptors in the vicinity from being activated by extracellular ATP. Our findings reveal an important role of human keratinocyte NTPDase2 in modulating nucleotide signaling in the extracellular milieu of human epidermis.

## Introduction

The skin is the interface between the human body and the external environment. It prevents water loss and forms a defensive barrier against various harmful stimuli [1]–[3]. The skin is also a sensory organ that is densely innervated by primary afferent sensory nerve endings [4]–[7]. The outermost layer of skin is a multilayer structure called the epidermis, where a variety of epidermal cells (keratinocytes, melanocytes, Langerhans cells, and Merkel cells) provide physical and immunological protection against trauma, microorganism invasion, and ultraviolet radiation [8]. Keratinocytes are the predominant cells in the epidermis, and originate in the basal skin layer and grow outward through the epidermis to replace dead keratinocytes that constantly shed from the skin surface.

Because of their primary function at the body-external environment interface, keratinocytes are prone to a variety of mechanical compressions and damage, which trigger wound healing, inflammation, and sensory stimuli transduction [9]. Emerging evidence underscores the importance of purine nucleotide signaling as a critical form of communication between cells of the skin, immune cells, and sensory neurons [10]. It has been shown that heat [11], hypo-osmotic shock [12], mechanical force [13], [14], and changing the culture media [15] can trigger adenosine triphosphate (ATP) release from the cytoplasm to extracellular space. In addition, when the epidermal layer is compromised by a wound or injury, concentrated ATP is released from the damaged skin cells.

ATP released from cells into the extracellular space has short half-life in the extracellular milieu before it is degraded to adenosine diphosphate (ADP), adenosine monophosphate (AMP), and adenosine, a process that in turn activates multiple receptors. There are currently seven known P2X ligand-gated ion channel receptor subtypes (P2X1-7) and eight P2Y G protein-coupled receptor subtypes (P2Y1, P2Y2, P2Y4, P2Y6, and P2Y11–14) for ATP and related nucleotides [16]–[18]. In the epidermis, P2 receptors are involved in regulating proliferation, differentiation, and apoptosis [19]–[21]. Nucleotide triphosphates and P2Y receptor activation protects HaCaT cells from H_2_O_2_-induced cell damage [22] and inhibits keratinocyte spreading and migration [23], and correlates with thermotransduction in skin [11], chemokine expression in human keratinocytes [24], interleukin (IL)-6 expression and release in normal human epidermal keratinocytes [25], and IL-6 production via intracellular calcium elevation [15], [26]. Human keratinocytes secrete nucleotide triphosphate and express multiple nucleotide receptors that correlate with cell proliferation, differentiation, apoptosis, and migration [27], [28].

Although much is known about the functional relationship between nucleotides and keratinocytes, how nucleotides are regulated after they are released into the extracellular space remains unclear. Several enzymes that hydrolyze extracellular nucleotides have been identified and characterized in various tissues [29]–[33]. The ectonucleoside triphosphate diphosphohydrolases (E-NTPDases) family represents the major nucleotide-hydrolyzing enzymes involved in purinergic signaling. These enzymes hydrolyze nucleotide tri- and diphosphates and sequentially convert them via ADP to AMP. Eight isoforms have been identified in mammals (NTPDase1–8), and four of them are surface-located enzymes that hydrolyze extracellular nucleotides. Despite nearly ubiquitous tissue expression, it has not been determined whether NTPDases are expressed in human keratinocytes [29], [32], [34].

In this study, we investigated the possibility that human keratinocytes express surface-located NTPDases that hydrolyze extracellular ATP and modulate P2Y receptor activation. HaCaT is a non-tumorigenic, spontaneously transformed human keratinocyte cell line [35] that was used as a model to determine whether keratinocytes release and hydrolyze ATP under static conditions. We used ATP bioluminescence, combined with siRNAs and an ecto-ATPase inhibitor, to identify functional NTPDase isoforms expressed in HaCaT cells. We also used fluorescence imaging, combined with siRNAs and a selective P2Y antagonist, to evaluate how NTPDases regulate P2Y receptor activation that is coupled to intracellular calcium ([Ca^2+^]_i_) mobilization. These results suggest that NTPDases might alter various P2Y receptor-mediated epidermal cellular functions through nucleotide conversion.

## Materials and Methods

### Reagents

ARL 67156, ATP, ADP, hexokinase, apyrase, and U73122 were purchased from Sigma. The ADP-Glo Kinase Assay was purchased from Promega. The siPORT NeoFX Transfection Agent, TRIzol Reagent, Hank's balanced salt solution (HBSS), HEPES, thapsigargin, Fluo-4 AM, CellTracker Blue CMAC, Fluo-4 NW Calcium Assay Kit, Probenecid, PowerLoad Concentrate, High Capacity cDNA Reverse Transcription Kits, TaqMan Fast Universal PCR Master Mix, and Silencer Select small interfering RNAs were purchased from Life Technologies. Femtotips II was purchased from Eppendorf. The ATP Bioluminescence Assay Kit CLS II and HS II were purchased from Roche.

### Cell culture

The HaCaT keratinocyte cell line [35] was provided by Dr. Te-Chang Lee (Institute of Biomedical Sciences, Academia Sinica, Taiwan). HaCaT cells were cultured in Dulbecco's modified Eagle medium (DMEM) supplemented with 10% fetal calf serum (FCS). Cells were incubated at 37°C in a humidified atmosphere of 95% air and 5% CO_2_. HaCaT cells were passaged at approximately 90% confluence and were incubated for 5 min in trypsin/EDTA. Cells were resuspended in fresh DMEM and seeded at a density of 6×10^5^ cells/10 cm culture dish.

### Small interfering RNA (siRNA) transfection

All transient transfection experiments were confirmed using one validated siRNA or at least two predesigned siRNAs and showed similar results. siRNAs (50 nM) specific for P2Y1 (siRNA ID: s9962), P2Y2 (s9967), NTPDase2 (s225158 and s2664), NTPDase3 (s2671 and s230383), NTPDase8 (s51786 and s51788), and control siRNAs (Silencer Select Negative Control #1 and #2) were transfected using the siPORT NeoFX Transfection Agent, according to the manufacturer's instructions. Cells being transfected were seeded at a density of 1.2×10^5^ cells/3.5 cm dish or 2×10^3^ cells/96-well plate. Forty-eight hours after siRNA transfection, the knockdown efficiency was verified by quantitative real-time polymerase chain reaction (PCR).

### RNA isolation and quantitative real-time PCR (qPCR)

RNA was isolated using the TRIzol Reagent according to the manufacturer's instructions. cDNA was synthesized from 2 µg total RNA using the High Capacity cDNA Reverse Transcription kit according to the manufacturer's instructions. qPCR was performed in 96-well format (in triplicate) on the StepOne Plus cycler (Applied Biosystems) using gene-specific TaqMan probes and TaqMan Fast Universal PCR Master Mix. The relative mRNA amount was calculated using 2^-^ΔΔ^Ct^ by normalizing to the housekeeping gene TATA-binding protein (TBP).

The TaqMan probes used were as follows: ENTPD1: Hs00169946_m1; ENTPD2: Hs00154301_m1; ENTPD3: Hs00154325_m1; ENTPD8: Hs01651150_m1; P2RY1: Hs00704965_s1; P2RY2: Hs00175732_m1 and Hs00602525_m1; P2RY4: Hs00267404_s1; P2RY6: Hs00173683_m1 and Hs00602547_m1; P2RY11: Hs01038858_m1; P2RY12: Hs00224470_m1; P2RY13: Hs00256749_s1; P2RY14: Hs00208434_m1; ENPP1: Hs01054040_m1; ENPP2: Hs00905125_m1; ENPP3: Hs01038393_m1; TBP: Hs99999910_m1. Most primers showed an amplification efficiency of 100% with less than a 3% error (Figure S1).

### ATP hydrolysis assays

HBSS supplemented with 20 mM HEPES (HBSS/HEPES) was used for all *in vitro* assays and measurements. All nucleotides were diluted to desired concentrations as 2X concentrates. Cells were cultured for 48 hr until confluence in transparent 96-well plates. The culture media was removed and cells were washed with 100 µl pre-warmed HBSS/HEPES prior to the addition of 50 µl HBSS/HEPEP or ARL 67156 (200 µM). The reaction was started by adding 50 µl of nucleotide solution to each well and then incubated for 1 hr at 37°C. Nucleotide samples were collected and placed on ice immediately for measurement.

### Extracellular ATP and ADP measurements

To measure ATP, samples were diluted 400×in water and measured in a 96-well format ATP Bioluminescence Assay Kit CLS II according to the manufacturer's instructions. The blank was subtracted from the raw data and ATP absolute concentrations were calculated from a standard curve of 10^−6^ to 10^−9^ M ATP. To measure ADP, the procedure was similar to ATP measurements except the samples were not diluted and were measured in a 384-well format ADP-Glo Kinase Assay. Absolute concentrations were calculated from a standard curve of 10^−4^ to 10^−7^ M ADP.

### Measurement of nucleotide-induced intracellular calcium increase

Forty-eight hours after siRNA transfection, cells were trypsinized, seeded at a density of 8.5×10^3^ cells in black 96-well plate, and grown for another 48 hr. Cells were washed once and incubated for 30 minutes at 37°C with Fluo-4 NW reagents. Intracellular calcium ([Ca^2+^]_i_) levels were monitored as changes in fluorescence intensity using a fluorescence plate reader equipped with an automatic dispenser (M200Pro, Tecan). A series of ATP or ADP solutions were applied, and fluorescence was recorded in parallel both before and after addition. When ADP was used as an agonist, 25 mM ADP was pretreated with 10 U hexokinase in the presence of 5mM glucose and 2.6 mM MgCl_2_ for 1 hr at 37°C to remove any trace of ATP before adding to cells. Each condition was performed in triplicate and repeated at least twice.

### Microscopy

To visually differentiate the two cell populations co-cultured in a cell monolayer, cells carrying the non-targeting control siRNA were labeled for 30 min at 37°C with 25 µM CellTracker Blue in OPTI-MEM 48 hr after siRNA transfection. The same labeling procedure was performed in parallel using vehicle (dimethyl sulfoxide) for the other population. The cells were trypsinized, mixed at 1:1 ratio, and cultured in a collagen-coated glass-bottom dish (Nest Biotechnology) for 48 hr. HaCaT cells labeled with CellTracker Blue showed identical response to cells without labeling (Figure S2).

HBSS/HEPES was used for all imaging experiments. To image intracellular calcium, cells were loaded with Fluo-4 AM (5 µM along with 2.5 mM Probenecid, 1X PowerLoad, and 1X NuclearMask Deep Red) for 30 minutes at room temperature. Imaging was performed using an inverted microscope (DM IRBE, Leica) equipped with a 20X dry objective (PLAN APO, 0.70 numerical aperture, Leica), a cooled charge-coupled device (CCD) camera (Cascade 512B, Photometrics), and mechanical shutters (Uniblitz). A microinjection glass needle (Femtotips II, Eppendorf) loaded with a 10 µM ATP solution was attached to a microinjection system (FemtoJet and InjectMan NI 2, Eppendorf), controlled by a custom-built Visual Basic program, to perform point release of ATP 3 µm above the cell surface at a defined time point. The injection pressure and duration were optimized to create calcium waves that touched the edge of the camera field of view (FoV) for each experiment. Green (Fluo-4), blue (CellTracker Blue), and infrared (NuclearMask Deep Red) images were acquired sequentially using MetaMorph 6.2 (Molecular Devices). Point release of ATP from the glass needle was visualized using Alexa Fluor 647 ATP in a separate experiment.

To visualize the mechanical force-induced ATP release event, the ATP Bioluminescence Assay Kit HS II was diluted 5-fold and added to a HaCaT cell monolayer. A solid glass needle was gradually lowered to apply pressure to one HaCaT cell until the bioluminescence signal was detected. Imaging was performed without any filter on an inverted microscope (DM IRB, Leica) equipped with a 20X oil-immersion objective (PLAN FLUOTAR, 0.75 numerical aperture, Leica) and a cooled CCD camera (ORCA-ER, Hamamatsu), located in a dark room.

### Image analyses

All image analyses were performed using MetaMorph 7.7 Offline (Molecular Devices) and Tableau Desktop 7.0 (Tableau). CellTracker Blue and NuclearMask Deep Red images were used to perform automatic cell demarcation and to separate two cell populations. Only calcium waves with 50±10% mixed cell populations are included in the analyses. Cells touching the edge of FoV and cells with centroids outside the cell boundaries were excluded. [Ca^2+^]_i_ response is presented as the fluorescence increase from baseline fluorescence (ΔF/F_0_). Cells with ΔF/F_0_ lower than 0.05 were defined as background fluctuation and were excluded. Calculation of the distance between the cell and microinjection needle is described and illustrated in Figure S3.

### Data processing and statistical analyses

Unless specified, all graphs are shown as the mean±standard error of the mean (SEM). When two groups were compared, a Student's *t*-test was used. Analysis of variance (ANOVA) was used to determine statistical significance. All data calculations and curve fittings were performed and graphed using Microsoft Excel and GraphPad Prism.

## Results

### HaCaT cells release ATP and hydrolyze extracellular ATP

**Figure 1:**
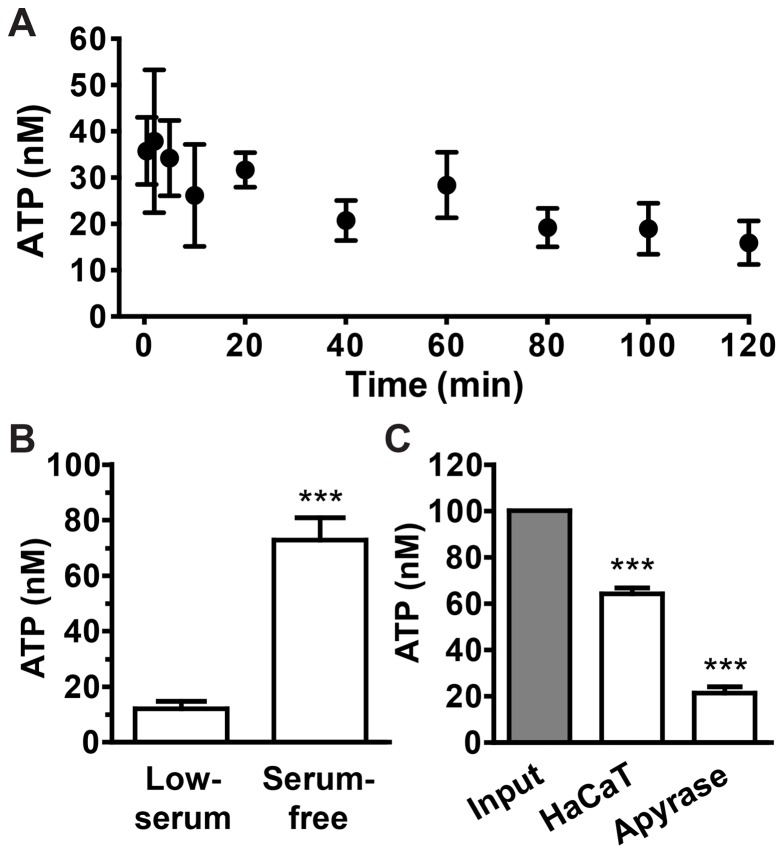
HaCaT cells release ATP and hydrolyze extracellular ATP. (A) Time course of basal ATP levels in the extracellular buffer after a media change. Data represent values from one representative experiment performed in triplicate (mean±SD). (B) ATP release from HaCaT cells into the extracellular space with (serum-free) and without (low-serum) the washing step to remove residual fetal calf serum (FCS). Bars denote ATP concentrations in the buffer after 1 hr in the presence of cells (mean±SEM, n = 16–17, Student's *t* test, *** *p*<0.001). (C) Hydrolysis of extracellular ATP (100 nM) in the presence of HaCaT cells. Apyrase (the Desiree isoform derived from red potatoes, 10^−3^ units/ml) serves as a positive control of ATP hydrolysis. Bars denote the concentration of remaining ATP compared with cell-free input (gray bar; mean±SEM, n = 5, one-way ANOVA with Dunnett's post-analysis, comparing all columns to cell-free input, *** *p*<0.001).

To determine whether HaCaT cells release ATP under static culture conditions, we replaced HaCaT culture media with fresh buffer and monitored the concentration of extracellular ATP in the presence of the cells. ATP can be detected in the extracellular buffer immediately after a media change, and the levels slowly dropped and remained constant after 40 minutes (Figure 1A). The average ATP concentration at 1 hr was approximately 12.1±4.9 nM (mean±SEM, Figure 1B). We noticed that the measured ATP concentration was significantly higher in culture wells that were washed once with fresh buffer than without washing, suggesting that culture media remained in the culture wells has ATP hydrolytic activity (72.8±33.3 nM, Figure 1B). We found that the different basal level of extracellular ATP was attributed to trace amounts of FCS, but not to secreted cellular factors in the culture media (Figure S4). Therefore, the complete removal of residual culture media by washing is required for the accurate determination of ATP concentrations.

To determine whether HaCaT cells hydrolyze extracellular ATP, we incubated cells in buffer containing defined amounts of ATP. As in the presence of apyrase, an enzyme that catalyzes ATP hydrolysis to AMP and inorganic phosphate, the ATP concentration is greatly reduced after 1 hr in the presence of HaCaT cells (Figure 1C). These data confirm HaCaT cells hydrolyze ATP in the extracellular milieu.

### HaCaT cells express surface-located NTPDases that digest ATP and accumulate ADP in the extracellular space

**Figure 2:**
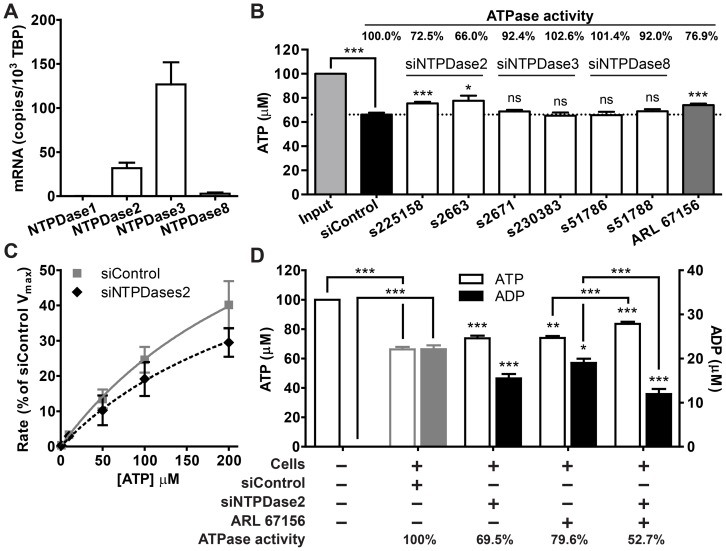
HaCaT cells express NTPDase2 that hydrolyzes extracellular ATP and accumulates ADP. (A) The relative mRNA levels of four surface-located NTPDases in HaCaT cells as determined using real-time PCR. Bars denote mRNA copies per 10^4^ TATA-binding protein (TBP) mRNA (mean±SEM, n = 2–21). (B) Hydrolysis of 100 µM ATP (light gray bar) when NTPDase expression is suppressed using siRNAs (white bars) or 100 µM ARL 67156, an NTPDases inhibitor (dark gray bar). ATPase activity is defined as the rate of ATP hydrolysis (amount of ATP hydrolyzed per hr) relative to the rate of control [mean±SEM, n = 2–21, one-way ANOVA with Dunnett's post-analysis, comparing all columns to control siRNA treatment (black bar), * *p*<0.05; *** *p*<0.001; ns = not significant]. (C) ATP hydrolysis kinetics in the presence of control or NTPDase2 siRNA-treated cells. Curves denote Michaelis-Menten kinetics normalized to the V_max_ of control cells (mean±SEM, n = 2). (D) Extracellular ATP hydrolysis and ADP accumulation when NTPDase2 was suppressed using siRNA and/or ARL 67156. Bars denote ATP (left Y-axis) and ADP (right Y-axis) concentrations compared to the respective controls (mean±SEM, n = 5, one-way ANOVA followed by Bonferroni's multiple comparison test, * *p*<0.05; ** *p*<0.01; *** *p*<0.001).

We evaluated the expression of four surface-located NTPDases (NTPDase1, 2, 3, and 8) using quantitative real-time PCR. NTPDase2 and 3 mRNAs were abundantly expressed whereas NTPDase1 and 8 mRNA levels were low or barely detectable in HaCaT cells (Figure 2A). To identify the NTPDase isoform that enables HaCaT cells to hydrolyze ATP, we suppressed NTPDase2, NTPDase3, and NTPDase8 expression using at least two isoform-specific siRNAs, and measured their respective ATP hydrolysis capabilities. Input ATP at a concentration of 100 µM was used to differentiate the digestion of exogenous ATP from spontaneously released ATP (usually <100 nM), which could contribute to significant variations if lower levels of input ATP were used. Although all NTPDase mRNAs were effectively knocked down, only siRNAs targeting NTPDase2 significantly affected ATP hydrolysis (Figures 2B and S5). In addition to siRNAs targeting NTPDases, the ecto-ATPase inhibitor ARL 67156, a competitive inhibitor of both human and mouse ATPases [36], markedly reduced ATP hydrolysis (Figure 2B). The hydrolysis rate versus substrate concentration relationship showed that the ecto-nucleotidase activity of HaCaT cells exhibit Michaelis-Menten kinetics and have a K_m_ of 303.1 µM. Knocking down NTPDase2 significantly lowered the V_max_ to 74% of control (Figure 2C).

The results presented thus far suggest that NTPDase2 plays a significant role in the hydrolysis of extracellular ATP in HaCaT cells. The substrate preferences and kinetic properties of NTPDases are currently thought to vary considerably among individual members [37]. We evaluated whether HaCaT cells generate sustainable levels of intermediates or hydrolyze ATP directly to AMP by monitoring ADP formation. Interestingly, along with ATP, a considerable amount of ADP can be detected at the end of the hydrolysis reaction (Figure 2D). Knocking down NTPDase2 expression using siRNA or inhibiting NTPDases activity with ARL 67156 simultaneously reduces ATP hydrolysis and ADP formation. The combined treatment of ARL 67156 and NTPDase2 siRNA synergistically affects ATP hydrolysis and ADP formation to a greater extent compared with ARL 67156 treatment alone. The amount of generated ADP is proportional to the amount of ATP hydrolyzed in all treatments, suggesting there is a positive correlation between NTPDases expression/activity and ADP formation (Figure 2D).

### ATP, not its hydrolyzed product, activates P2Y receptors and mobilizes intracellular calcium

**Figure 3:**
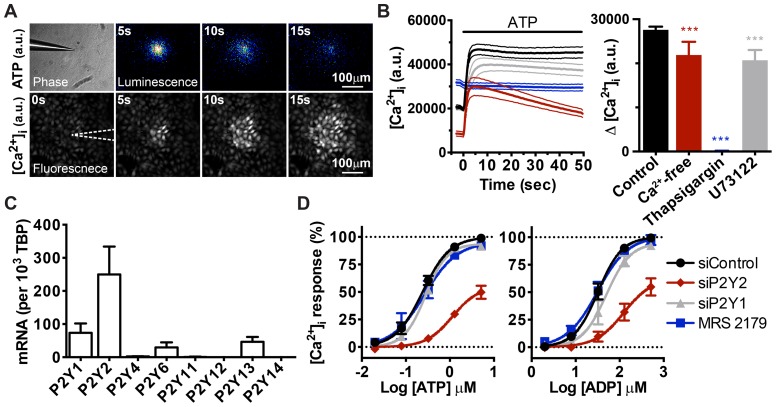
ATP evokes [Ca^2+^]_i_ increase in HaCaT cells via P2Y2 receptor activation. (A) Visualization of extracellular ATP and intracellular calcium when one HaCaT cell was activated by mechanical force. Images represent montages of ATP bioluminescence (upper panel) and intracellular calcium fluorescence (lower panel) after touching a cell with a glass needle. White dotted lines outline the position of glass needle (a.u., arbitrary unit). (B) The kinetics (left) and amplitude (right) of the [Ca^2+^]_i_ increase when HaCaT cells were pre-incubated in Ca^2+^-free HBSS (red line), 1 µM thapsigargin (blue line), 10 µM U73122 (gray line), or untreated (black line), and stimulated with 5 µM ATP. Dotted lines indicate SD. Bars denote the amplitudes of the increases in the left panel (mean±SEM, n = 8, one-way ANOVA followed by Dunnett's multiple comparison test, *** *p*<0.001, a.u., arbitrary unit). (C) The relative expression levels of eight P2Y receptor mRNAs in HaCaT cells, as evaluated using real-time PCR. Bars denote the mRNA levels expressed as copies per 10^3^ TBP mRNAs. (D) ATP- and ADP-evoked [Ca^2+^]_i_ increases in HaCaT cells in which P2Y1 and P2Y2 were knocked down using siRNA or inhibited using the P2Y1-selective antagonist MRS 2179 (10 µM). Sigmoid curves denote the dose-response relationships of peak [Ca^2+^]_i_ increases to various concentrations of ATP and ADP. Values are normalized to the maximal response of siControl (mean±SEM, n = 2 to 5).

Mechanical force applied to the cell membrane results in membrane deformation and triggers immediate ATP release in human keratinocytes [12], [13], [38]. We detected an ATP release event when a HaCaT cell was gently touched with a glass needle (Figure 3A, upper panel). Such mechanical force also elicited a transient increase in [Ca^2+^]_i_ in the cells being touched as well as adjacent cells, forming a symmetrical pattern known as calcium wave (Figure 3A, lower panel). The similarity between the ATP diffusion pattern and calcium wave spreading upon mechanical stimulation suggested that ATP triggers [Ca^2+^]_i_ increase. In fact, the direct application of ATP to HaCaT cells is sufficient to elicit an instant increase in [Ca^2+^]_i_ (Figure 3B, left panel). Such a response was significantly suppressed when phospholipase C was inhibited by U73122 and completely abolished if calcium was depleted from internal store by thapsigargin, indicating that ATP mobilized intracellular calcium through the G protein-coupled P2Y receptor pathway. Additionally, ATP triggered an almost equal [Ca^2+^]_i_ increase irrespective of the presence of extracellular calcium, suggesting that P2Y is the dominant calcium-mobilizing ATP receptor in HaCaT cells (Figure 3B, right panel).

To identify which nucleotide receptors HaCaT cells express, we used quantitative real-time PCR to examine the mRNA levels of eight P2Y nucleotide receptor subtypes. Multiple P2Y subtype mRNAs were detected in HaCaT cells, including G_q_-coupled, [Ca^2+^]_i_-mobilizing P2Y1 and P2Y2 that are activated by ADP and ATP, respectively (Figure 3C). Because HaCaT cells release ATP and generate ADP, we reasoned that both P2Y2 and P2Y1 potentially contribute to the extracellular ATP-induced [Ca^2+^]_i_ increase.

To elucidate which receptor is involved in the mobilization of intracellular calcium, we first determined the sensitivity of HaCaT cells to both ATP and ADP. The dose-response curve of the ATP-evoked [Ca^2+^]_i_ increase had an EC_50_ of 240.4 nM [205.2 to 281.7 nM, 95% confidence interval (CI)], whereas the response curve to ADP had an EC_50_ of 32.2 µM (25.6 to 40.5 µM, 95% CI). The two curves only partially overlapped in the lower micromolar range, suggesting that HaCaT cells generate ADP that evokes subtle [Ca _i_ increases by an ATP concentration that already maximizes [Ca^2+^]_i_ increases in HaCaT cells (Figure 3D). Knocking down P2Y2 using siRNA markedly reduced the response, justifying the role of P2Y2 in mediating the ATP-induced [Ca^2+^]_i_ increase (Figure 3D). In contrast, both P2Y1 knockdown and MRS 2179, a P2Y1 antagonist, did not significantly alter the ATP-induced [Ca^2+^]_i_ response, excluding ADP receptor participation in the ATP concentration range sufficient to saturate [Ca^2+^]_i_ increase in HaCaT cells (Figure 3D). These results confirm that low micromolar to submicromolar ATP levels evoke [Ca^2+^]_i_ increases predominantly via ATP rather than its hydrolyzed product ADP and that both the endogenous P2Y2 receptor and intracellular calcium can be used to monitor ATP signaling at the cellular level.

**Figure 4:**
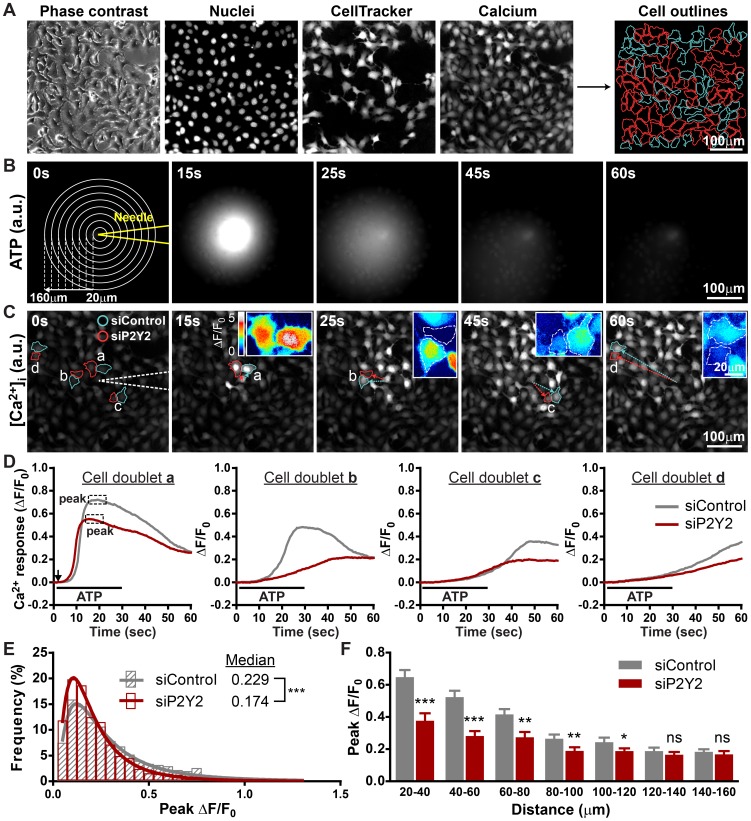
Knocking down P2Y2 expression attenuates the ATP-mediated [Ca^2+^]_i_ increase. (A) Fluorescence images showing how software-assisted cell demarcation and population identification is performed according to DNA and cell tracer dye staining. Cyan lines define control cells, and red lines outline P2Y2-deficient cells. (B) Time series of fluorescence imaging of Alexa Fluor-647 ATP released from microinjection needle. Concentric circles define the radial distances from needle tip in 20 µm steps (a.u., arbitrary unit). (C) Time series of fluorescence imaging of calcium wave spreading in a co-culture monolayer containing an equal ratio of control and P2Y2-deficient HaCaT cells. Four representative cell doublets with various distances from the needle are outlined in cyan and red. White dotted lines outline the position of glass needle. Insets: enlarged pseudo-color images of control and P2Y2-deficient cell doublets depicted in ΔF/F_0_. Dotted lines depict the cell outline (a.u., arbitrary unit). (D) Traces showing Ca^2+^ change (fold change from baseline, ΔF/F_0_) in cell doublets from panel C. The black arrow indicates the initiation of ATP point release, and the black line defines the duration of ATP point release. (E) Frequency distribution and log Gaussian fit comparing the peak Ca^2+^ change between control (gray) and P2Y2-deficient (red) cells. (832 vs. 953 cells pooled from 12 calcium waves, Mann-Whitney test was used to determine statistical significance). (F) Bar graph comparing the average of median peak [Ca^2+^]_i_ response between control and P2Y2-deficient cells within the same range relative to the needle tip (mean±SEM, n = 12 calcium waves, Student's *t* test, * *p*<0.05; ** *p*<0.01; *** *p*<0.001; ns = not significant).

To further verify the role of P2Y2 in transmitting extracellular nucleotide signals, we compared the kinetics of ATP-induced [Ca^2+^]_i_ increase between wild-type and P2Y2-deficient HaCaT cells. Cells transfected with non-targeting control siRNA and anti-P2Y2 siRNA were co-cultured at 1∶1 ratio and stimulated with the same ATP gradient (Figure 4A). To simulate intercellular ATP signaling initiated from a single cell, a micromanipulator and injector were programmed to semi-automatically release the ATP solution near the cell surface (Figure 4B). Point release of a defined amount of ATP solution from a glass needle positioned a few micrometers above the cell surface creates a symmetrical calcium wave, which is similar to that triggered by mechanical force (Figure 4C). The diffused ATP gradient elicits a symmetrical but heterogeneous calcium wave with patches of cells showing attenuated [Ca^2+^]_i_ increase in the mixed monolayer culture (Figure 4C). Inspection of single-cell [Ca^2+^]_i_ kinetics from pairs of P2Y2-expressing and -deficient cells with equal distance relative to the glass needle shows that the response of P2Y2-deficient cells is lower than P2Y2-expressing cells, as judged by the peak amplitude of [Ca^2+^]_i_ increase after ATP addition (Figure 4D). Statistical analyses of the two populations revealed that the heterogeneity in the ATP-induced calcium waves correlates with P2Y2 expression. The histogram of peak responses was shifted to the left in the P2Y2-deficient population, and the average calcium response of P2Y2-deficient cells is significantly lower than that of P2Y2-expressing wild-type cells across calcium wave range, confirming the role of P2Y2 in triggering [Ca^2+^]_i_ increase (Figure 4E and 4F).

### NTPDase2 locally regulates P2Y receptor activation and attenuates ATP-induced [Ca^2+^]_i_ mobilization

**Figure 5:**
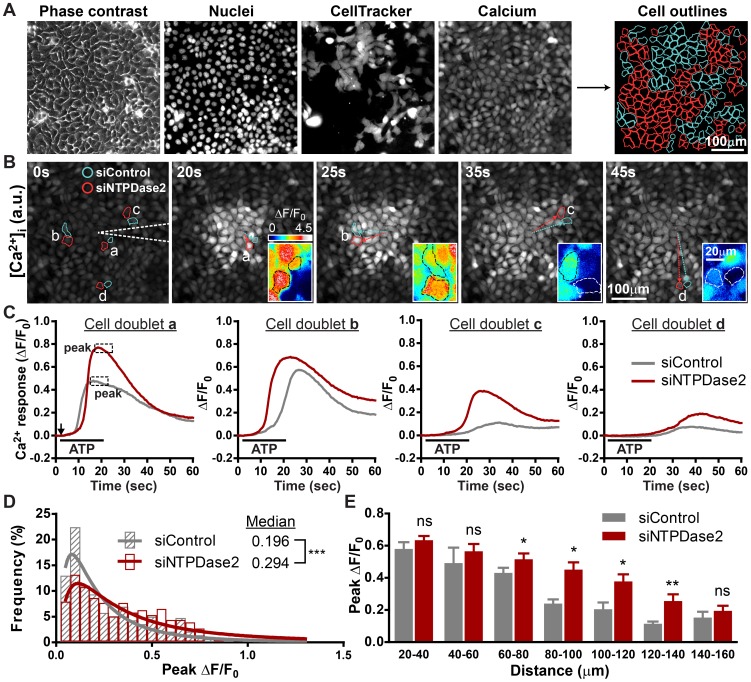
Knocking down NTPDase2 locally potentiates ATP-induced [Ca^2+^]_i_ increase. (A) Fluorescence images showing how software-assisted cell demarcation and population identification is performed according to DNA and cell tracer dye staining. Cyan lines define control cells, and red lines outline P2Y2-deficient cells. (B) Time series of fluorescence imaging of calcium wave spreading in a co-culture monolayer containing an equal ratio of control and NTPDase2-deficient HaCaT cells. White dotted lines indicate the position of the glass needle. Insets: enlarged pseudo-color images of control and NTPDase2-deficient cell pairs depicted in ΔF/F_0_. Dotted lines define the cell outline (a.u., arbitrary unit). (C) Traces showing Ca^2+^ change (fold change from baseline, ΔF/F_0_) in individual control (gray) and NTPDase2-deficient (red) cells from panel B. The black arrow indicates the initiation of ATP point release, and the black line defines the duration of ATP point release (a.u., arbitrary unit). (D) Frequency distribution and log Gaussian fit comparing the peak Ca^2+^ change between control (gray) and NTPDase2-deficient (red) cells. (414 vs. 422 cells pooled from 5 calcium waves, Mann-Whitney test was used to determine statistical significance). (E) Bar graph comparing the average median peak [Ca^2+^]_i_ response between control and NTPDase2-deficient cells within the same range relative to the needle tip (mean±SEM, n = 5 calcium waves, Student's *t* test, * *p*<0.05; ** *p*<0.01; ns = not significant).

To determine whether NTPDase2 controls the accessibility of extracellular ATP to its receptor near the cell surface, the ATP-induced [Ca^2+^]_i_ responses of wild-type and NTPDase2-deficient cells were compared when both populations were stimulated with the same ATP gradient (Figure 5A). HaCaT cells transfected with control siRNA and anti-NTPDase2 siRNA were mixed and cultured to form a confluent monolayer. Point release of ATP induced similar [Ca^2+^]_i_ kinetics in both cell populations. However, the peak [Ca^2+^]_i_ responses of NTPDase2-expressing cells are lower than those of NTPDase2-deficient cells (Figure 5B and 5C). The histogram of peak [Ca^2+^]_i_ increase revealed that the median response of NTPDase2-expressing cells is significantly lower than NTPDase2-deficient cells, consistent with our hypothesis that the presence of NTPDase2 on the HaCaT cell surface limits the accessibility of ATP to its receptor and attenuates local ATP-induced [Ca^2+^]_i_ increase (Figure 5D). To examine NTPDase2-mediated ATP signaling in greater detail, the [Ca^2+^]_i_ responses of both cell populations at various distances from the glass needle were compared. Whereas most NTPDase2-expressing cells at distal areas show lower mean peak responses than NTPDase2-deficient cells, the responses of both populations near the glass needle (where they were stimulated with higher ATP doses) do not show a significant difference, suggesting that NTPDase2 has limited ATP-hydrolyzing capacity (Figure 5E).

## Discussion

We have shown that HaCaT cells spontaneously release and hydrolyze ATP. ATP released into the extracellular space activates P2Y2 receptors and triggers [Ca^2+^]_i_ increase. In the presence of NTPDase2, extracellular ATP is hydrolyzed and converted into ADP. Our observations also indicate that in addition to modulating ATP concentration in the pericellular microenvironment, NTPDase2 functions as a local signal suppressor that shields adjacent P2Y2 receptors from being accessed by the primary messenger (extracellular ATP) and thus attenuates activation of the secondary messenger (intracellular calcium).

Previous results have shown that cultured human keratinocytes release ATP under static conditions [39], [40]. ATP can be released via several possible routes, such as membrane channels and exocytosis [41]–[44]. However, the exact mechanism of ATP release in human keratinocytes has not been well defined. Interestingly, we noticed that in the presence of residual serum, the level of extracellular ATP is insufficient to initialize [Ca^2+^]_i_ mobilization (Figures 1B and 3D). However, in the absence of serum, ATP rose to a level that is within the dose-response curve of ATP-induced [Ca^2+^]_i_ increase. These observations suggest that in typical culture conditions, serum in the culture media metabolizes extracellular ATP to levels below the threshold needed to trigger the [Ca^2+^]_i_ response and masks any potential NTPDase activity. Therefore, the indispensability of serum for cell growth and its high ATP-hydrolyzing activity might obscure the effects of ecto-nucleotidase and must be considered in *in vitro* experiments studying the physiological role of ecto-nucleotidase.

Because HaCaT cells digest extracellular ATP, we reasoned that they express ecto-nucleotidase on their cell surface. We provide multiple lines of evidence indicating that NTPDase2 is the major NTPDase isoform responsible for ecto-nucleotidase activity in HaCaT cells. First, NTPDase2 mRNA is abundantly expressed in HaCaT cells (Figure 2A). Second, according to current knowledge, the substrate preferences and kinetic properties of NTPDases vary considerably among individual members. Although NTPDase1 converts ATP directly to AMP without producing any intermediates, other NTPDases produce a sustained (NTPDase2) or transient (NTPDases3 and 8) accumulation of ADP [37]. By monitoring ATP consumption and ADP formation, we found that HaCaT cells accumulate ADP in the presence of ATP, consistent with the kinetic properties of NTPDase2 (Figure 2D). Third, transient NTPDase2 gene silencing significantly reduced ATP hydrolysis as well as ADP formation (Figures 2B-D, S5). Finally, although NTPDase3 and NTPDase8 mRNAs were detected and readily suppressed by siRNAs, ATP hydrolysis was not perturbed (Figures 2B and S5). The accumulation of ADP also indicates that NTPDase3 and NTPDase8 might not be functionally expressed or may only play minor roles. Taken together, our data support the notion that NTPDase2 is the major NTPDase isoform accounting for extracellular ATP hydrolysis in HaCaT cells; the roles of NTPDase isoforms 3 and 8 have yet to be identified.

Our analyses demonstrate that NTPDase2 enables monolayers of HaCaT cells to reduce the concentration of ATP solution from the range of tens of nanomolar to hundreds of micromolar. However, unlike *in vitro* experiments conducted with the cell monolayer bathed in bulk solution, cells *in vivo* grow in a dense tissue matrix and release limited amounts of signaling molecules that rapidly diffuse into a confined space in which nearby cells are stimulated by the signal gradient. Therefore, measuring ATP hydrolysis in bulk solution might underestimate the actual activity at the cell surface [45]. The ATP concentration inside a cell is typically 1 to 10 mM [46]. A K_m_ of 303.1 µM, measured from the hydrolysis of ATP in solution, implies that, in the presence of limited amounts of released ATP, surface-located NTPDases lower the overall ATP concentration in the pericellular microenvironment as well as instantaneously hydrolyze ATP and block local receptors from ATP stimulation. If this were the case, interfering with NTPDase expression would be expected to alter the local ATP-induced cellular response. Consistent with our hypothesis, our recordings of single-cell [Ca^2+^]_i_ dynamics show that NTPDase2 expression correlates with attenuation of the ATP-induced [Ca^2+^]_i_ increase on the same cell, although with limited capacity (Figure 5).

NTPDases are known to regulate intercellular signaling in various tissues [47]. Although an attempt to fuse P2Y1 and NTPDase1 had been described [48], data supporting the concept that endogenous NTPDases co-localize with P2Y receptors and differentially regulate P2Y signaling are scarce [49]–[52]. Our observation indicates that NTPDase2 shields P2Y receptors in close proximity from being accessed by nucleotide ligands. Further information regarding the ratio and spatial arrangement between specific NTPDase isoforms and P2Y subtypes would help delineate how NTPDases modulate P2Y activation in the membrane microdomain.

We have shown that the ATP-induced [Ca^2+^]_i_ increase exhibits a typical sigmoid dose-response relationship, indicating that the amplitude of [Ca^2+^]_i_ increase reflects the ATP dosage to which the cells are exposed (Figure 3D). Therefore, we used intracellular calcium, a secondary messenger that is downstream of ATP, as a reporter to determine whether NTPDases regulate ATP-induced cellular response. By releasing ATP solution from a microinjection needle, we can control the dosage and duration of stimulation and thus create ATP-induced calcium waves more consistently than by applying a mechanical force to the cell membrane. In addition, using this method, steep ATP gradients are achieved and one can find cells stimulated by various ATP concentrations in a single calcium wave. Furthermore, stimulating a mixed culture comprised of two cell populations avoids variations between the treatments to two separate cultures.

HaCaT cells express P2Y1 and P2Y2, G_q_-coupled receptors that elicit [Ca^2+^]_i_ increase upon activation by ADP and ATP, respectively. Because HaCaT cells accumulate ADP in the presence of ATP, we speculated that the ATP-induced [Ca^2+^]_i_ increase is triggered synergistically by P2Y1 and P2Y2. Due to the lack of selective antagonists, we were not able to pharmacologically suppress the P2Y2 receptor. Additionally, it has been shown that some P2 receptor antagonists inhibit NTPDases [53], [54]. Using P2Y subtype-specific siRNAs and a P2Y1 selective antagonist, we confirmed the participation of P2Y2 receptor and found that ADP and P2Y1 do not contribute to [Ca^2+^]_i_ increase when cells are activated by micro- to sub-micromolar amounts of ATP (Figure 3D). The results from NTPDase2-deficient cells also excludes the participation of ADP and defines NTPDase2 as a regulator that suppresses ATP signaling, rather than promotes ADP signaling, in mobilizing [Ca^2+^]_i_ (Figure 5D and 5E).

Although ADP triggers [Ca^2+^]_i_ increases in HaCaT cells, such responses were not suppressed by P2Y1 siRNA or MRS 2179 treatment (Figure 3D, right panel). Interestingly, responses were more profoundly affected by ATP receptor suppression. The similarity between the two panels in Figure 3D suggests that ATP might be involved in ADP-induced [Ca^2+^]_i_ increases. The addition of hexokinase, an enzyme that converts ATP into ADP in the presence of glucose, to the extracellular buffer efficiently suppressed the ADP-induced [Ca^2+^]_i_ increase in a dose-dependent manner (Figure S6). This finding indicates that HaCaT cells use ADP as a substrate to generate ATP at a level that is sufficient to activate the P2Y2 receptor (Figures S6 and 3D). It has been reported that human keratinocytes are capable of generating ATP at the cell surface, although the exact mechanism and the physiological phosphate donor are both unknown [40]. Another possibility is that micromolar levels of ADP stimulate ATP secretion in a dose-dependent manner. Although the nature of this phenomenon is unclear, the concept of ADP-induced ATP generation or secretion could possibly explain the insensitivity of HaCaT cells to P2Y1 suppression and elucidate why ADP induces [Ca^2+^]_i_ increases at an EC_50_ 134-times higher than ATP.

In addition to the NTPDase family, there are other surface-located enzymes that have been identified that hydrolyze extracellular ATP, such as the ectonucleotide pyrophosphatase/phosphodiesterase (E-NPP) family, ecto-5'-nucleotidase/CD73, and alkaline phosphatases [31], [33]. In fact, we detected the mRNAs of all NPP isoforms (NPP1, NPP2, and NPP3) in HaCaT cells, but the expression levels were very low (data not shown). Furthermore, NPPs do not accumulate ADP in the presence of ATP, suggesting that NPPs are not the major ATP-hydrolyzing enzymes in HaCaT cells. In this report, we characterized NTPDase2 as the modulator of intercellular ATP signaling, and the contribution of other enzymes to extracellular ATP hydrolysis remains to be determined.

HaCaT cells also express P2Y13, a G_i_-coupled ADP receptor that inhibits adenylyl cyclase and decreases intracellular cyclic AMP (cAMP) when activated by nanomolar amounts of ADP [55], [56]. Because NTPDase2 accumulates ADP, the possible role of P2Y13 in perturbing intracellular cAMP in HaCaT cells is of particular interest. In addition to adenine nucleotide receptors, mRNAs of uracil nucleotide receptors were also detected in HaCaT cells, including P2Y2, P2Y4, and P2Y6 (Figure 3C). Uridine-5'-triphosphate (UTP) has been shown to stimulate interleukin-6 production in HaCaT keratinocytes and modulate keratinocyte migration via P2Y receptor activation [15], [23], [26], [57], [58]. Our preliminary results confirm that both UTP and uridine diphosphate (UDP) can elicit [Ca^2+^]_i_ increase, suggesting functional uracil nucleotide receptors are expressed in HaCaT keratinocytes (unpublished data). Because all surface-located NTPDases dephosphorylate UTP with significant accumulation of UDP [37], NTPDase expression on the keratinocyte plasma membrane would be expected to regulate uracil nucleotide-mediated re-epithelialization during would healing.

Taken together, the results suggest that when HaCaT cells are in a resting state or are activated by membrane distortion, limited amounts of ATP are released into the extracellular space, which rapidly diffuses and is hydrolyzed by NTPDase2 into ADP. Unlike ATP that immediately activates P2Y2 receptors and transiently elevates [Ca^2+^]_i_, ADP generated by HaCaT cells does not reach sufficient levels to elevate [Ca^2+^]_i_. However, in certain pathophysiological conditions when cells are exposed to large amounts of ATP, NTPDases and ecto-5'-nucleotidase produce a variety of primary signals, such as ADP, AMP, and adenosine, and the activation of a multiplicity of membrane receptors results in secondary signals that promote cell proliferation, differentiation, and migration.

In conclusion, we described nucleotide hydrolyzing enzyme expression on the surface of a human keratinocyte cell line and provided data regarding how they modulate activation of receptors in the vicinity and orchestrate intracellular secondary messenger mobilization from the pericellular microenvironment. Given the position and role of keratinocytes in the skin, understanding nucleotide metabolism as well as autocrine/paracrine signaling between various skin cells and sensory neurons would benefit further research regarding normal epidermal homeostasis and wound healing.

## Supporting Information

Figure S1 **Amplification efficiencies of qPCR primers.** The PCR amplification efficiency of each TaqMan assay was measured using the C_t_ slope method with at least three data points (concentrations) covering a 4-log dilution range of HaCaT cDNA. Amplification efficiency (Ex) was calculated from the slope using the equation: Ex = 10^(-1/slope)^ – 1. The Ex and the coefficient of determination (R^2^) of each slope were indicated using matching colors.
(TIF)

Figure S2 **Long-term CellTracker Blue labeling does not interfere with ATP-induced [Ca^2+^]_i_ increase.** (A) Frequency distribution and log Gaussian fit comparing the peak Ca^2+^ change between CellTracker-free (gray) and CellTracker-containing (red) cells. (394 vs. 454 cells pooled from 5 calcium waves, Mann-Whitney test was used to determine statistical significance). (B) Bar graph comparing the average of median peak [Ca^2+^]_i_ response between CellTracker-free (gray) and CellTracker-containing (red) cells within the same range relative to the needle tip (mean±SEM, n = 5 calcium waves, Student' *t* test, ns = not significant).
(TIF)

Figure S3 **Illustration showing the calculation of the relative distance between a cell and the tip of the glass needle.** Because the distance from the needle to the nearest cell edge cannot be precisely determined by image analysis software, the approximate distance is calculated from the mean radius of the cell.
(TIF)

Figure S4 **Serum has high ATPase activity and hydrolyzes ATP in a dose-dependent manner.** Bars denote the concentration of remaining ATP when 100 nM ATP was incubated with DMEM, FCS, or conditioned medium (CM, collected from a 48-hr culture) at the indicated volumes in the absence of HaCaT cells at 37°C for 60 min. FCS was pre-diluted to 10% in DMEM to be comparable to the serum level in CM [mean±SEM, n = 5, two-way ANOVA followed by Tukey's multiple comparison test comparing all columns to mock treatment (solid bars), *** *p*<0.001; ns = not significant].
(TIF)

Figure S5 **The relative mRNA levels 96** **hr after gene-specific siRNA knockdown.** Two pre-designed siRNAs were used to verify gene-specific knockdown of NTPDase2, NTPDase3, and NTPDase8. P2Y1 and P2Y2 were knocked down using one validated gene-specific siRNA. Values are normalized to non-targeting control siRNA (dark gray bar, mean±SEM, n = 3 to 12, one-way ANOVA with Dunnett's post-analysis comparing all columns to non-targeting control siRNA, *** *p*<0.001).
(TIF)

Figure S6 **Extracellular hexokinase suppresses the ADP-induced [Ca^2+^]_i_ increase.** Bars denote the nucleotide-induced peak [Ca^2+^]_i_ increase with or without extracellular hexokinase that was added right before the assay. Numbers on the gray bar denote the amount of hexokinase in a 150 µl reaction volume (mean±SEM, n = 3, RFU: relative fluorescence unit defined as ΔF/F_0_, two-way ANOVA followed by Bonferroni's multiple comparison test, ** *p*<0.01; *** *p*<0.001; ns = not significant).
(TIF)
