# Supplementary figures and images for: Ecto-Nucleoside Triphosphate Diphosphohydrolase 2 Modulates Local ATP-Induced Calcium Signaling in Human HaCaT Keratinocytes
Source: PLoS One. 2013 Mar 11;8(3):e57666. doi: 10.1371/journal.pone.0057666 (PMC3594229; doi:10.1371/journal.pone.0057666)

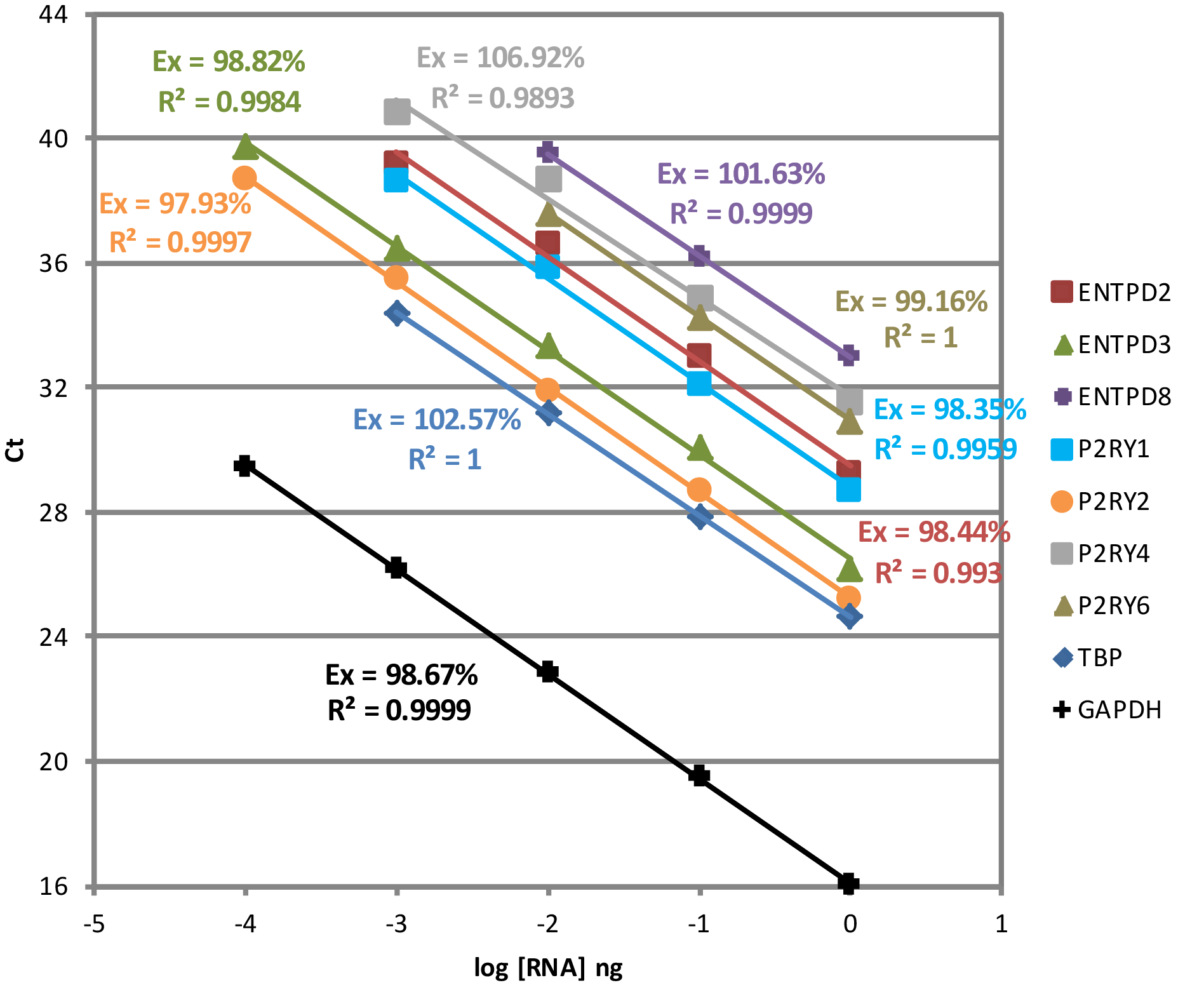

Supplement: Figure S1 — Amplification efficiencies of qPCR primers. The PCR amplification efficiency of each TaqMan assay was measured using the Ct slope method with at least three data points (concentrations) covering a 4-log dilution range of HaCaT cDNA. Amplification efficiency (Ex) was calculated from the slope using the equation: Ex = 10(-1/slope) – 1. The Ex and the coefficient of determination (R2) of each slope were indicated using matching colors. (TIF) [file pone.0057666.s001.tif]

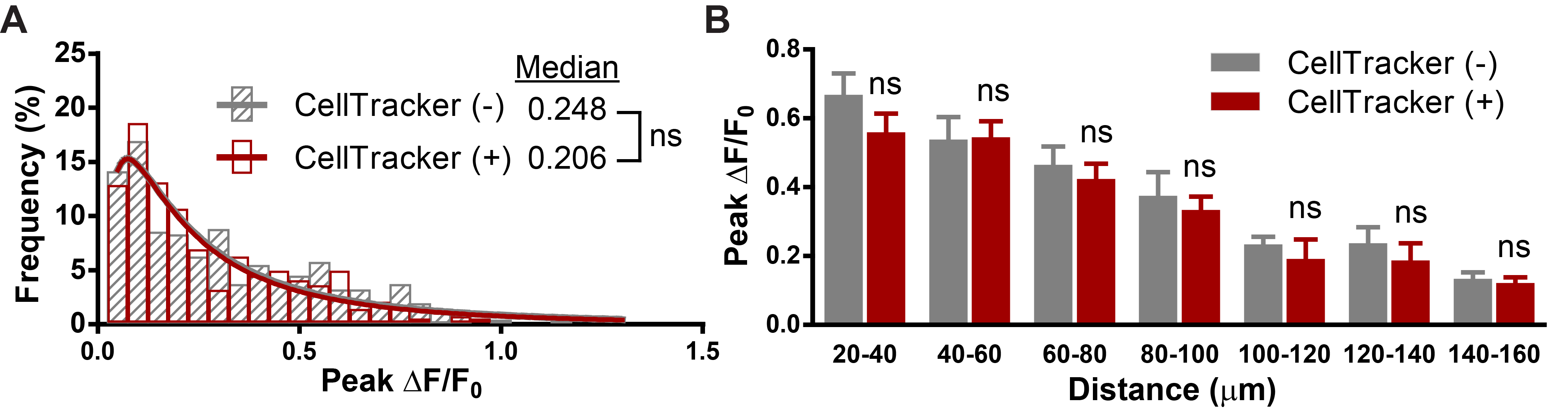

Supplement: Figure S2 — Long-term CellTracker Blue labeling does not interfere with ATP-induced [Ca2+]i increase. (A) Frequency distribution and log Gaussian fit comparing the peak Ca2+ change between CellTracker-free (gray) and CellTracker-containing (red) cells. (394 vs. 454 cells pooled from 5 calcium waves, Mann-Whitney test was used to determine statistical significance). (B) Bar graph comparing the average of median peak [Ca2+]i response between CellTracker-free (gray) and CellTracker-containing (red) cells within the same range relative to the needle tip (mean±SEM, n = 5 calcium waves, Student' t test, ns = not significant). (TIF) [file pone.0057666.s002.tif]

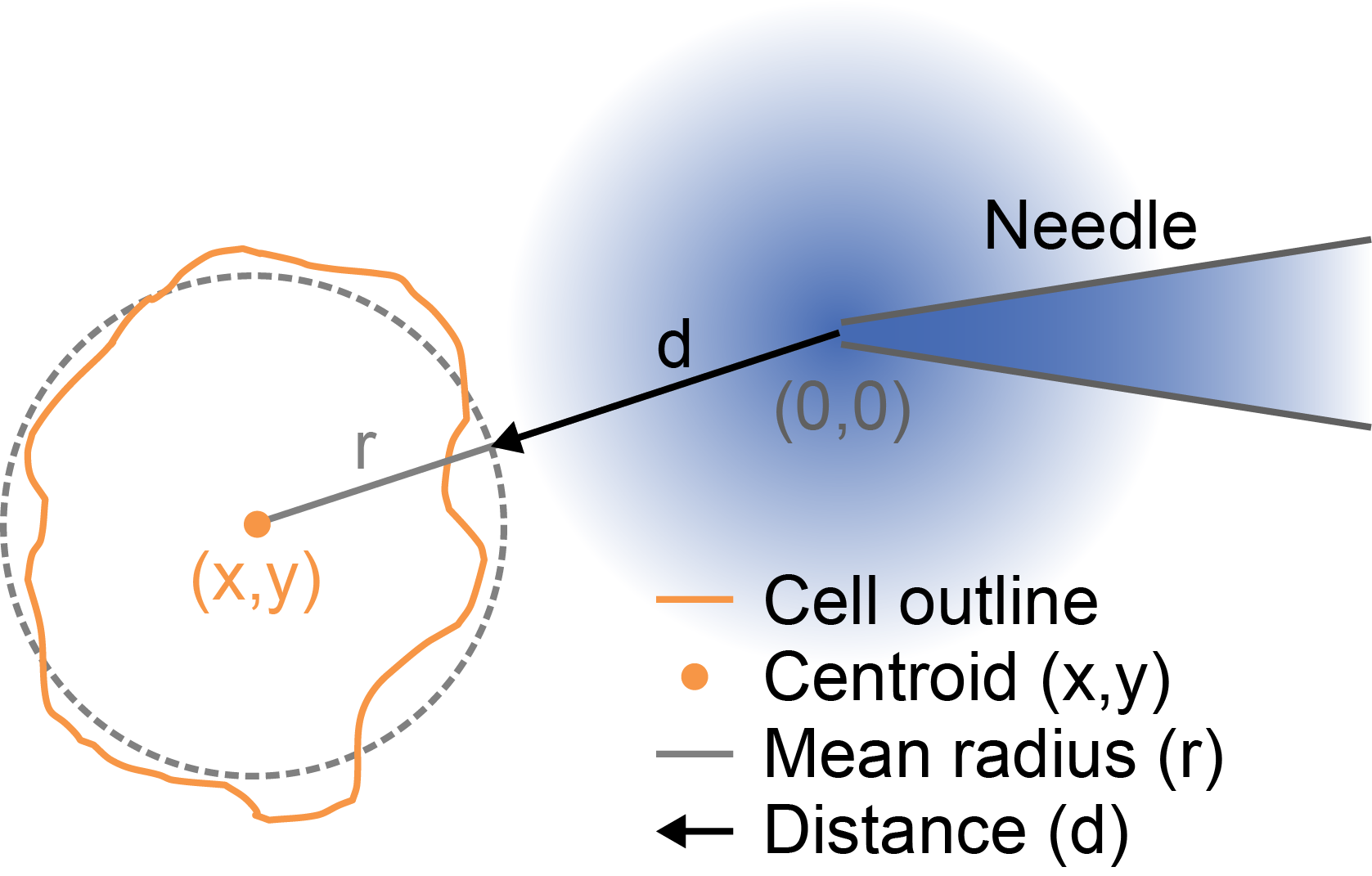

Supplement: Figure S3 — Illustration showing the calculation of the relative distance between a cell and the tip of the glass needle. Because the distance from the needle to the nearest cell edge cannot be precisely determined by image analysis software, the approximate distance is calculated from the mean radius of the cell. (TIF) [file pone.0057666.s003.tif]

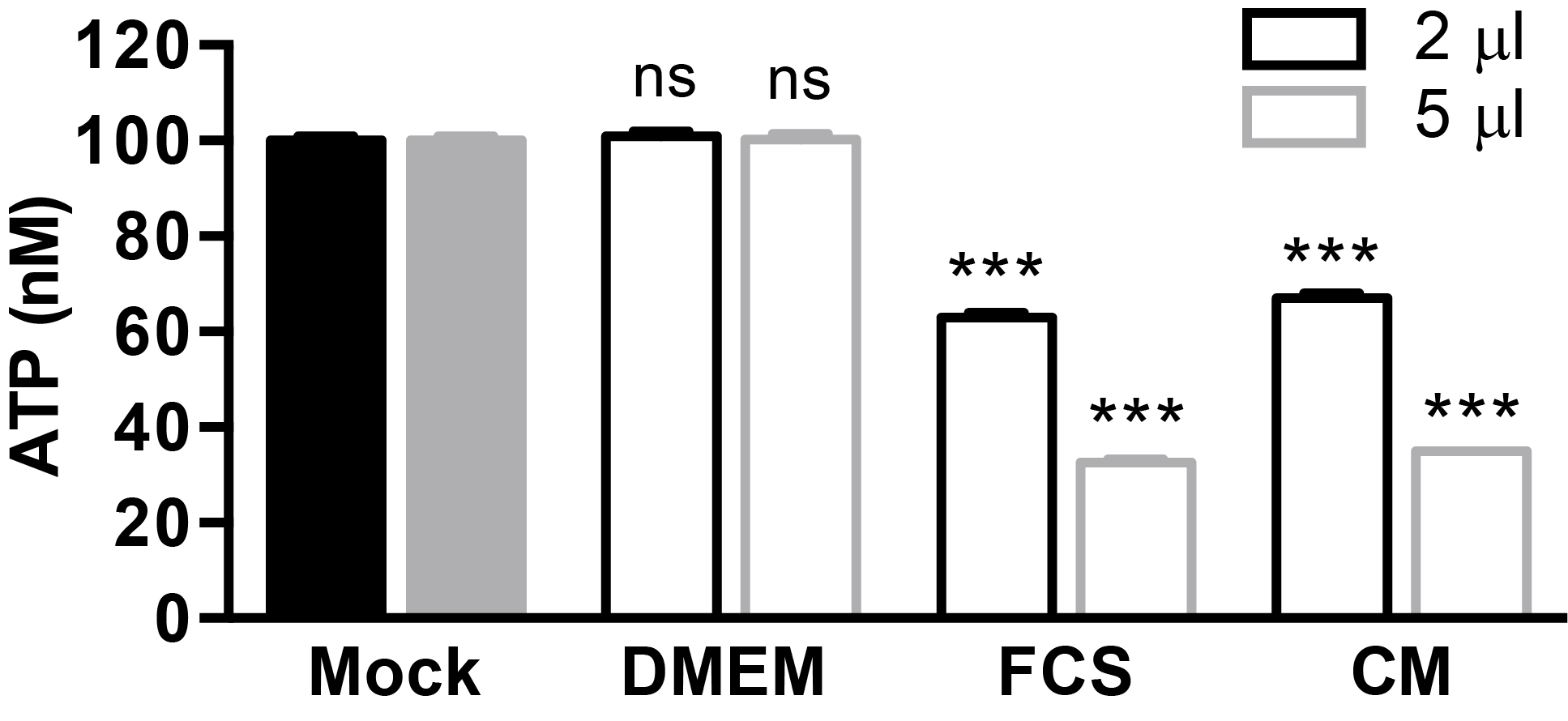

Supplement: Figure S4 — Serum has high ATPase activity and hydrolyzes ATP in a dose-dependent manner. Bars denote the concentration of remaining ATP when 100 nM ATP was incubated with DMEM, FCS, or conditioned medium (CM, collected from a 48-hr culture) at the indicated volumes in the absence of HaCaT cells at 37°C for 60 min. FCS was pre-diluted to 10% in DMEM to be comparable to the serum level in CM [mean±SEM, n = 5, two-way ANOVA followed by Tukey's multiple comparison test comparing all columns to mock treatment (solid bars), *** p<0.001; ns = not significant]. (TIF) [file pone.0057666.s004.tif]

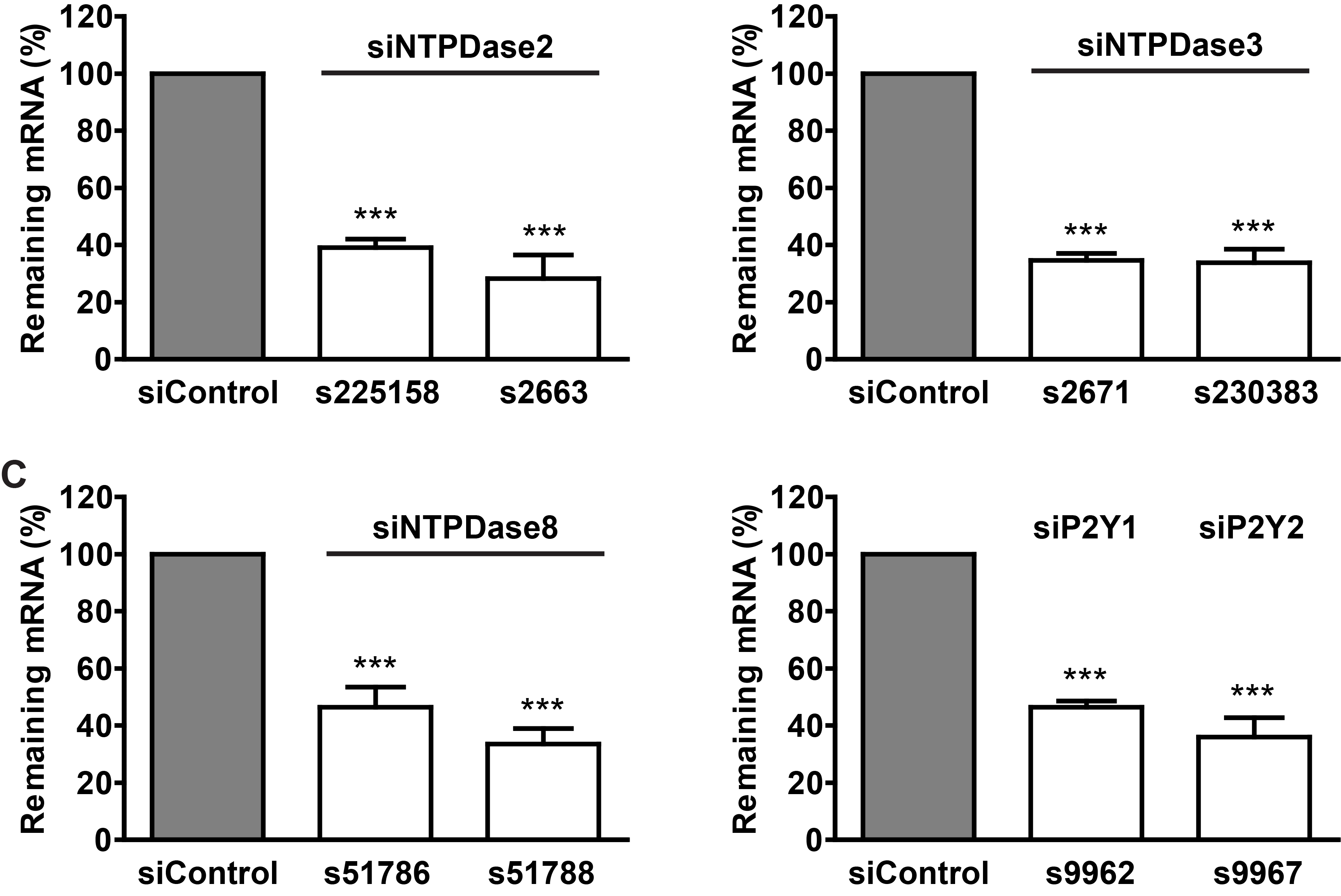

Supplement: Figure S5 — The relative mRNA levels 96 hr after gene-specific siRNA knockdown. Two pre-designed siRNAs were used to verify gene-specific knockdown of NTPDase2, NTPDase3, and NTPDase8. P2Y1 and P2Y2 were knocked down using one validated gene-specific siRNA. Values are normalized to non-targeting control siRNA (dark gray bar, mean±SEM, n = 3 to 12, one-way ANOVA with Dunnett's post-analysis comparing all columns to non-targeting control siRNA, *** p<0.001). (TIF) [file pone.0057666.s005.tif]

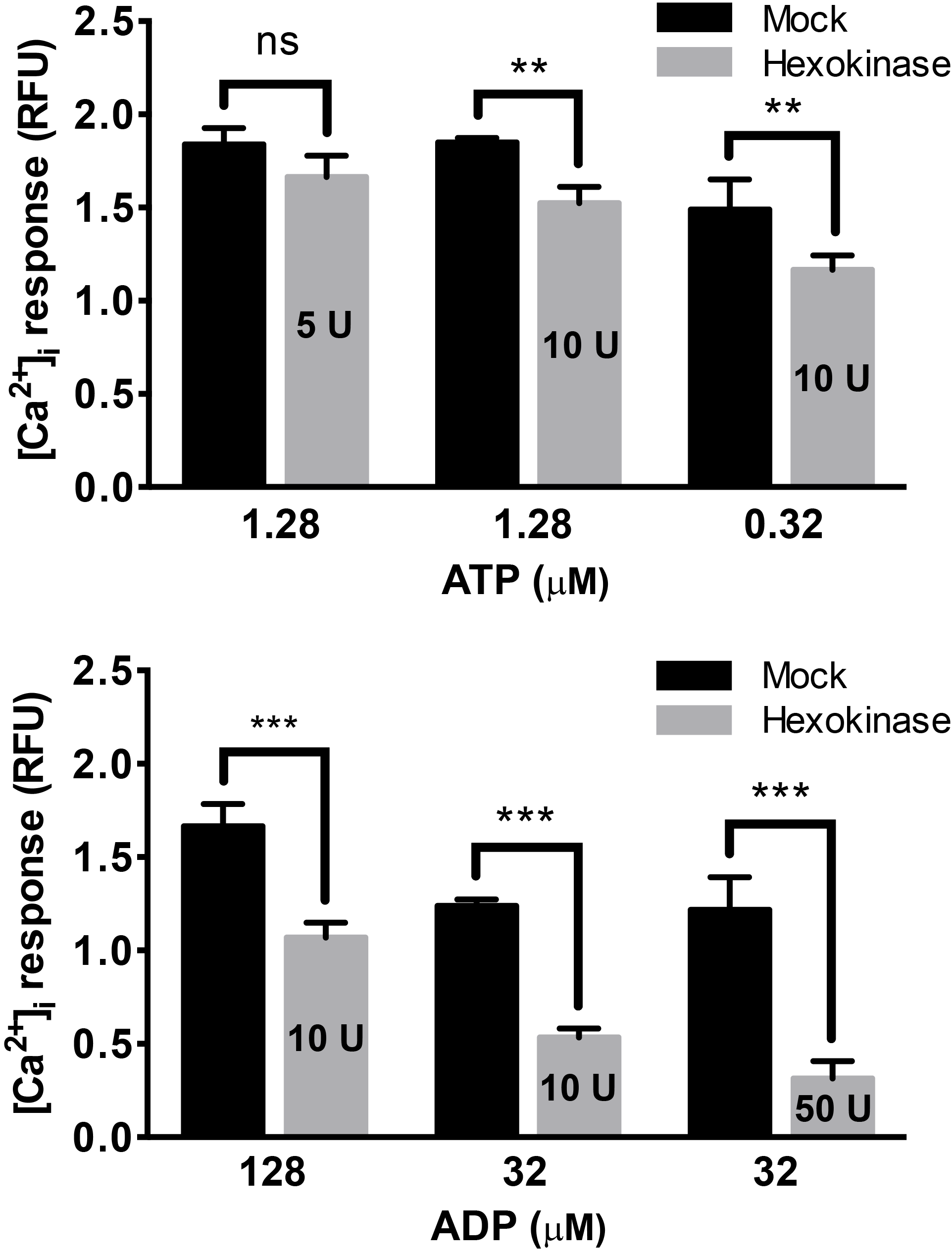

Supplement: Figure S6 — Extracellular hexokinase suppresses the ADP-induced [Ca2+]i increase. Bars denote the nucleotide-induced peak [Ca2+]i increase with or without extracellular hexokinase that was added right before the assay. Numbers on the gray bar denote the amount of hexokinase in a 150 µl reaction volume (mean±SEM, n = 3, RFU: relative fluorescence unit defined as ΔF/F0, two-way ANOVA followed by Bonferroni's multiple comparison test, ** p<0.01; *** p<0.001; ns = not significant). (TIF) [file pone.0057666.s006.tif]
